# Transforming literature screening: The emerging role of large language models in systematic reviews

**DOI:** 10.1073/pnas.2411962122

**Published:** 2025-01-06

**Authors:** Fernando M. Delgado-Chaves, Matthew J. Jennings, Antonio Atalaia, Justus Wolff, Rita Horvath, Zeinab M. Mamdouh, Jan Baumbach, Linda Baumbach

**Affiliations:** ^a^Institute for Computational Systems Biology, Faculty of Mathematics, Informatics and Natural Sciences, University of Hamburg, Hamburg 22761, Germany; ^b^Center for Motor Neuron Biology and Diseases, Department of Neurology Columbia University, New York, NY 10032; ^c^Inserm Center of Research in Myology, Neuro-Myology Service G.H. Pitié-Salpêtrière, Sorbonne Université, Paris 75013, France; ^d^Syte – Strategy Institute for DigitalHealth, Hamburg 20354, Germany; ^e^Department of Clinical Neurosciences, University of Cambridge, Cambridge CB2 0QQ, United Kingdom; ^f^Department of Pharmacology and Personalised Medicine, Maastricht University, Maastricht 6229 ER, The Netherlands; ^g^Department of Pharmacology and Toxicology, Faculty of Pharmacy, Zagazig University, Zagazig 44519, Egypt; ^h^Department of Mathematics and Computer Science, Institute for Mathematics and Computer Science, University of Southern Denmark, Odense 5230, Denmark; ^i^Department of Health Economics and Health Services Research, University Medical Center Hamburg-Eppendorf, Hamburg 20246, Germany; ^j^Center for Bioinformatics Hamburg, Faculty of Mathematics, Informatics and Natural Sciences, University of Hamburg, Hamburg 22761, Germany

**Keywords:** large language models, systematic reviews, literature screening

## Abstract

Systematic reviews (SR) synthesize evidence-based medical literature, but they involve labor-intensive manual article screening. Large language models (LLMs) can select relevant literature, but their quality and efficacy are still being determined compared to humans. We evaluated the overlap between title- and abstract-based selected articles of 18 different LLMs and human-selected articles for three SR. In the three SRs, 185/4,662, 122/1,741, and 45/66 articles have been selected and considered for full-text screening by two independent reviewers. Due to technical variations and the inability of the LLMs to classify all records, the LLM’s considered sample sizes were smaller. However, on average, the 18 LLMs classified 4,294 (min 4,130; max 4,329), 1,539 (min 1,449; max 1,574), and 27 (min 22; max 37) of the titles and abstracts correctly as either included or excluded for the three SRs, respectively. Additional analysis revealed that the definitions of the inclusion criteria and conceptual designs significantly influenced the LLM performances. In conclusion, LLMs can reduce one reviewer´s workload between 33% and 93% during title and abstract screening. However, the exact formulation of the inclusion and exclusion criteria should be refined beforehand for ideal support of the LLMs.

## Significance

Large language models (LLMs) can effectively be utilized for prefiltering scientific records for systematic reviews, leading to a substantial reduction in manual workload. Their performance in selecting scientific records based on titles and abstracts depends on the interplay between the inclusion and exclusion criteria and the LLM. Therefore, refining the formulation of the inclusion and exclusion criteria with the support of the LLM prior to title and abstract screening has the potential to improve the LLM’s performance in selecting relevant records.

A systematic literature review (SR) summarizes existing knowledge and provides the highest source of evidence regarding specific research questions. Its results indicate research gaps, highlight the quality of evidence by incorporating “Grading of Recommendations, Assessment, Development, and Evaluations” (1), and are used to inform clinical recommendations. However, performing a high-quality SR following scientific guidelines, like the Preferred Reporting Items for SRs and Meta-Analyses (PRISMA) is time-consuming (2). Crucial early steps involve formulating the research question, defining inclusion and exclusion criteria, setting up a search strategy, and selecting articles for inclusion.

It is critical to establish clear inclusion and exclusion criteria for articles in order to properly address the specific research question at hand. These inclusion criteria are subsequently integrated into a comprehensive search matrix, which is applied across diverse databases and sources. This systematic procedure aims to yield a collection of all potentially relevant articles, which frequently exceeds several thousand scientific articles even after duplicate removal. In accordance with the accepted standard for the study selection procedure, two independent researchers in the field must screen this collection of articles. Researchers judiciously employ the predefined inclusion and exclusion criteria in a dual-stage assessment, initially limiting their scrutiny to the titles and abstracts (“screening 1”) and subsequently encompassing the full texts (“screening 2”). In the instance of disagreement regarding the inclusion of a certain article, the researchers either discuss or involve an additional researcher to come to a conclusion. This screening process for study inclusion is notably laborious, typically demanding several working days for each researcher involved.

Keeping up with the current state of clinical research is difficult due to its volume and variety of sources and formats, which can easily result in information overload (3). Attempting to stay up to date with new research becomes more difficult due to the exponential rise in medical articles, which is fueled by the “publish-or-perish” mentality and widespread internet access (4). An article estimated that the doubling time of medical knowledge was 50 y in 1950, 7 y by 1980, 3.5 y in 2010, and projected to be just 73 d in 2020 (5). This rapid expansion means that what was learned in the first 3 y of medical school becomes only a small fraction of the knowledge available by the end of the decade.

AI is a promising field to support researchers in their work. Specifically, large language models (LLMs) like the GPT family are advanced AI systems capable of understanding and generating human-like text based on vast amounts of training data (6). These models can help process extensive literature more quickly than manual methods, potentially reducing the time researchers need to review publications, though the accuracy and reliability of their outputs require careful human verification. However, LLMs face a significant limitation in their context window, restricting the amount of text they can process simultaneously (7), which poses challenges when dealing with lengthy scientific articles, particularly in SRs.

To overcome this limitation, researchers have developed the retrieval-augmented generation (RAG) framework (8). RAG combines retrieval-based methods with generative models, enabling LLMs to access and process information beyond their limited context windows. In this approach, a retrieval system first identifies the most relevant sections from a large corpus using vector search techniques. These relevant sections are then fed into the LLM, allowing it to generate responses based on a broader context than its inherent limitations would allow. RAG’s ability to handle large volumes of text makes it particularly suitable for SRs, enhancing tasks such as article identification, selection, and summarization and potentially streamlining the SR process while maintaining high methodological quality.

The use of LLMs in SRs is increasingly explored to streamline literature screening and enhance the reliability of evidence synthesis. Nashwan and Jaradat (9) demonstrate LLMs’ utility in automating quality assessments and risk-of-bias evaluations, traditionally time-consuming and subjective tasks. Similarly, Hasan et al. (10) propose a structured framework for GPT-4 in risk of bias assessments within SRs, delving into a specific application of LLMs in bias evaluation.

Applications of LLMs in screening and selection processes are also being investigated. Our approach extends this utility to automate the initial screening of titles and abstracts, providing a direct measure of efficacy by comparing LLM outputs with human decisions. In ref. 11, authors discuss potential applications in clinical trials, emphasizing the need for robust validation frameworks, directly relevant to our methodology, which compares LLM outputs against human benchmarks. Moreover, Wang et al. (12) explore zero-shot LLMs for SR screening, focusing on fine-tuning and calibration techniques.

Performance evaluation and validation of LLMs in SR tasks are critical. Hasan et al. (10) use performance metrics to quantify each model’s capability to accurately replicate human decision-making, potentially reducing reviewer workload. Khraisha et al. (13) investigate the potential to replace human input in SRs with GPT-4, focusing on its multi-language capabilities and extraction accuracy. Our research builds on this by assessing the generalizability and efficiency of multiple LLMs across different architectures and datasets. In contrast to studies focusing on fine-tuning and calibration, our study evaluates multiple LLMs against predefined inclusion and exclusion criteria, directly comparing their outputs to human decisions without recalibrating the models based on initial outputs. This approach not only showcases the capabilities of various LLMs in handling SR tasks but also emphasizes the importance of refining selection criteria to optimize LLM integration into the SR process.

The aim of this paper is to evaluate the reliability of LLMs in selecting all relevant articles based on title and abstracts (screening 1) for SRs. We utilized lists of identified articles from three existing SRs, feeding them separately to the LLMs. Subsequently, we compared the included and excluded articles identified by 18 different LLMs with those determined by the authors. This study examines whether the use of LLMs, particularly through the RAG framework, can streamline the SR study selection process. Our investigation into the influence of defined inclusion and exclusion criteria on LLM performance addresses a key challenge in leveraging LLMs for SRs, demonstrating how the criteria-model interplay can significantly impact screening accuracy. By testing 18 LLMs on datasets from three preexisting SRs across diverse medical topics, our approach enhances the generalizability of our findings and provides a deeper understanding of LLM consistency and robustness in handling various research questions and literature bases.

## Results

An overview of the included and excluded articles by humans for the three reviews on a physiotherapeutic, a neurologic, and a digital health topic, respectively, is provided in Table 1. Due to the exclusion of duplicates, articles published in languages other than English, and technical variations, the number of records fed to the LLMs was lower than the number of articles originally screened in the original SRs. The models used for the analyses are detailed in Table 5.

**Table 1.** Overview of data preprocessing together with ground truth (human officers)

	Review I (Physio)		Review II (Neuro)		Review III (DigiHealth)	
	Original	Preprocessed	Original	Preprocessed	Original	Preprocessed
Total number of records	4,662	4,501	1,741	1,650	66	66
Passed Screening 1	185	181	122	113	45	45
Passed Screening 2	75	73	34	22	6	6

Analysis of processing completeness revealed distinct patterns across the three SRs. In Review I (Physio, 4,501 records), most models achieved near-complete coverage, with only minor processing gaps: GPT-3.5-turbo (4 missing), GPT-4-mini (2 missing), Gemma-7b (6 missing), Gemma2-9b (5 missing), llama3.1-70b (4 missing), and llama3-Athene-70b (5 missing). Reviews II (Neuro) and III (DigiHealth) demonstrated perfect processing completion, with all 18 models successfully analyzing their complete datasets of 1,650 and 66 records, respectively.

When requiring all set inclusion criteria to be rated as “true” for a positive screening decision, we found that the average number of correctly classified titles and abstracts as either included or excluded were 4,294 (92%, min 4,130; max 4,329), 1,539 (88%, min 1,449; max 1,574), and 27 (40%, min 22; max 37) for the three reviews, respectively. Corresponding confusion matrices, illustrating these performances, are presented in *SI Appendix*, Figs. S1–S3. The matrices account for variations in the number of processed articles, as some models were unable to classify certain records, leading to slight differences in the total number of evaluated articles across models. However, the presented percentage respects these records, as they have to be evaluated by a second human researcher as those articles with conflicting classification between an LLM and the first human reviewer.

**Fig. 1.**
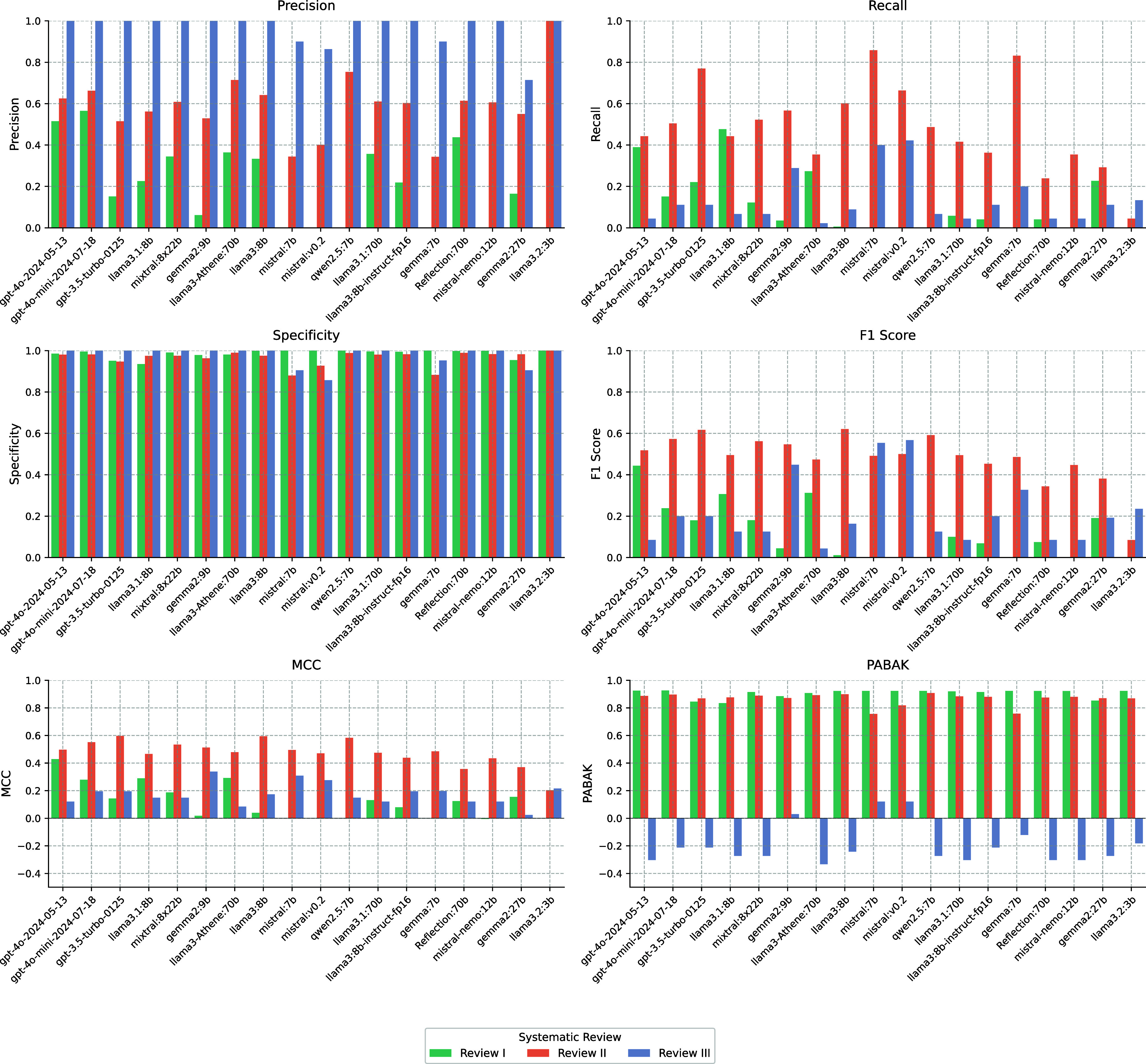
Comparison of model performance across multiple metrics, including precision, recall, specificity, F1 score, MCC, and PABAK, for different review sets (I Physio, II Neuro, III Digital Health). Models are ranked based on mean MCC, highlighting their relative performance in terms of robustness and reliability. MCC and PABAK are used to compare model performance, considering the effects of class imbalance and chance agreement. The color-coded bars represent the LLMs’ performance within the three SRs we evaluated.

In Fig. 1, the comparative analysis of different models across multiple evaluation metrics offers a comprehensive perspective on their performance and robustness. The models were assessed using precision, recall, specificity F1 score, Matthews correlation coefficient (MCC), and PABAK. These metrics gauge the models’ capabilities in identifying true positives, minimizing false positives, and ensuring balanced decision-making. The results are presented in bar plots, with models ranked according to their mean MCC, a particularly robust metric for scenarios involving imbalanced datasets.

The evaluation of model performance revealed notable differences among the LLMs. Based on MCC, the top-performing models were gpt-4o-2024-05-13 (mean MCC = 0.349), gpt-4o-mini-2024-07-18 (mean MCC = 0.342), and gpt-3.5-turbo-0125 (mean MCC = 0.312). GPT-4’s superior MCC performance indicates its robust ability to balance true positives and false positives, particularly important given the inherent class imbalance in SRs. While commercial GPT models led the rankings, open-source alternatives demonstrated competitive performance, with llama3.1:8b (mean MCC = 0.302) and mixtral:8x22b (mean MCC = 0.290) rounding out the top five performers. Notably, models with larger parameter counts did not necessarily perform better, as evidenced by the strong performance of smaller models like llama3.1:8b compared to its 70B counterpart (mean MCC = 0.242).

When examining the prevalence-adjusted bias-adjusted kappa (PABAK), which assesses agreement while accounting for class imbalance, we observed a different ranking of top performers. Mistral:v0.2 achieved the highest PABAK (0.621), followed by mistral:7b (0.600) and gemma2:9b (0.596). The strong performance of Mistral models in PABAK suggests their particular strength in agreement with human reviewers when adjusting for prevalence and bias. Notably, several smaller models outperformed their larger counterparts, with llama3.2:3b (mean PABAK = 0.537) and qwen2.5:7b (mean PABAK = 0.520) achieving higher scores than larger models like llama3.1:70b (mean PABAK = 0.500) and gemma2:27b (mean PABAK = 0.483). This pattern suggests that model size may not be the primary determinant of screening performance when accounting for class imbalance.

Several models demonstrated strong performance across both metrics. GPT-4o-mini-2024-07-18 emerged as one of the most consistent performers, combining high MCC (0.342) with solid PABAK performance (0.537). The Mistral family of models exhibited strong capabilities, with mistral:v0.2 achieving the highest PABAK score (0.621) and mistral:7b following closely (mean PABAK = 0.600). Gemma2:9b demonstrated robust performance across both measures (mean MCC = 0.290, PABAK = 0.596), indicating reliable screening capabilities. Notably, the low correlation between MCC and PABAK scores (0.053) suggests these metrics capture different aspects of model performance, highlighting the importance of considering both when evaluating models for SR screening tasks.

Looking at the SR-specific results (Reviews I, II, and III), we observed varying performance patterns across different models. The visualization of performance metrics reveals that models generally achieved higher specificity compared to recall, reflecting the challenge of identifying relevant articles in imbalanced datasets. This variation in performance across different reviews suggests that model selection should consider the specific characteristics and requirements of the SR being conducted.

## Additional Analyses

**Fig. 2.**
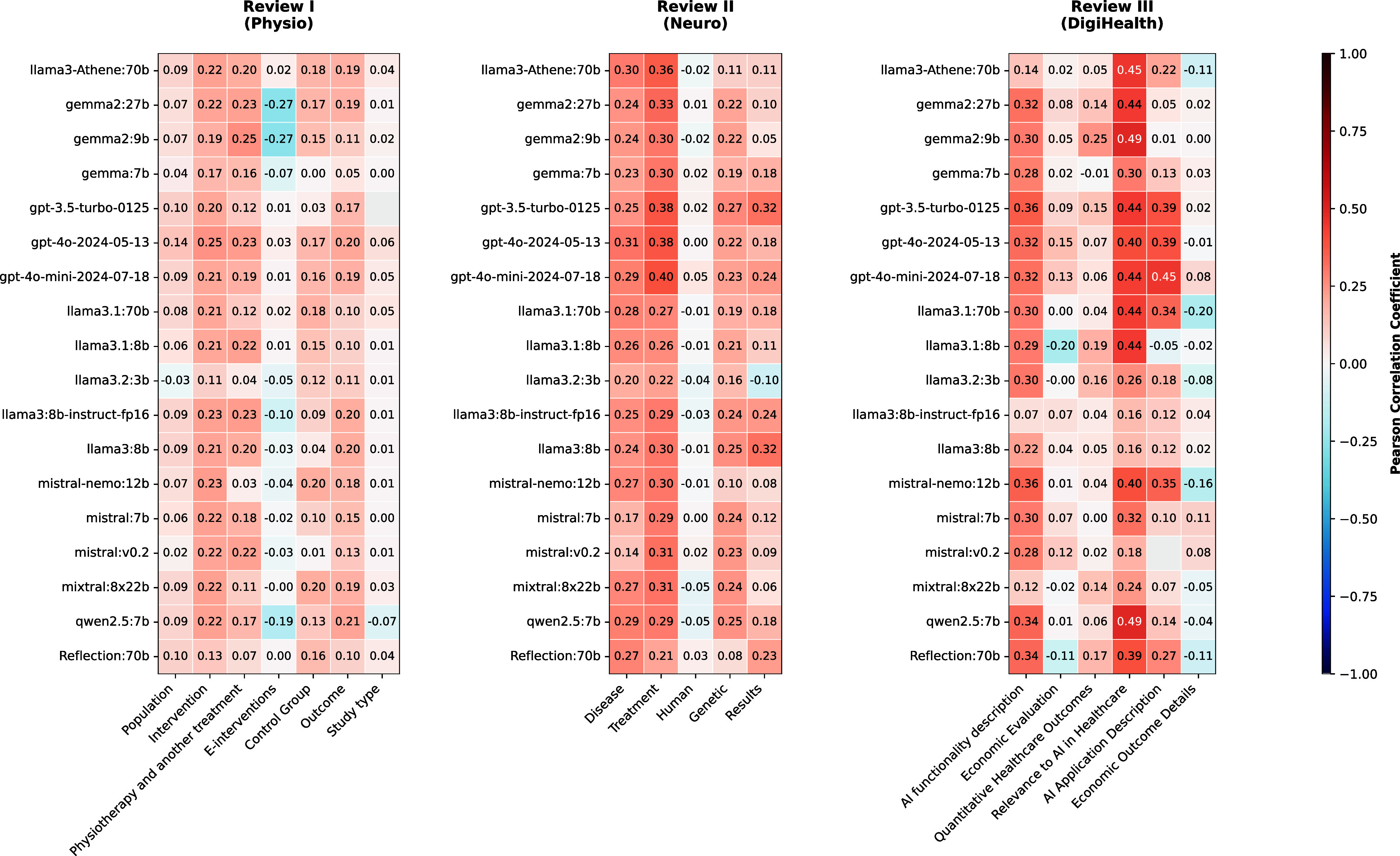
Pearson correlation coefficients between Boolean values for the criteria, predicted by different LLMs, and the Target Variable screening 1 for Reviews I, II, and III. Each heatmap represents one of the three distinct reviews, with LLMs listed on the y-axis and Boolean criteria on the *x*-axis. The color intensity, ranging from blue (negative correlation) to red (positive correlation), indicates the strength and direction of the linear relationship between each feature and screening 1.

We investigated the relationships between the inclusion and exclusion criteria predicted by LLMs and the target variable (screening 1) across our three reviews using Pearson’s correlation coefficient. The correlation analysis revealed that positive correlations were more prominent, indicating that certain criteria consistently showed a significant positive relationship with the screening decision. This highlights the potential of these criteria to serve as reliable indicators for inclusion. The correlations are visualized using heatmaps, as shown in Fig. 2.

Correlation analysis across the three systematic reviews revealed distinct patterns in criteria assessment. Review I (Physio) exhibited weak to moderate positive correlations (0.1 to 0.25) across most criteria, with physiotherapy treatment and intervention assessments showing the most consistent correlations across models. Notably, e-interventions showed appropriate negative correlations (up to −0.27 for gemma2:27b and gemma2:9b), correctly identifying studies to exclude. Review II (Neuro) demonstrated stronger associations, particularly in disease and treatment criteria where GPT models achieved correlations up to 0.40. Review III (DigiHealth) showed the highest correlations among all reviews, with models consistently achieving strong correlations (0.40 to 0.49) in assessing AI healthcare relevance.

The heatmap visualization helps identify key criteria that LLMs rely on when predicting screening 1, thus offering valuable insights into their decision-making processes. These insights provided some foundation for further analysis to work on and refine the inclusion and exclusion criteria. As a supplementary example, we explored the effectiveness of using a Random Forest (RF) classifier to select articles predicted as screening 1 true/false rather than relying solely on articles where all criteria are true. Here, we found that the number of true positive classified articles increased (*SI Appendix*, Figs. S4–S6). However, the number of true negative records dropped. Consequently, the mean percentage of correctly classified records dropped for the physiotherapeutic Review I from 92% to 74%, was similar with 87% compared to 88% in the neurological Review II, and increased from 40% to 65% in Review III on digital health. Further model performances are shown in *SI Appendix*, Fig. S7. Overall, the RF approach improved performance metrics, as evidenced by the higher MCC and PABAK values. The RF model was able to balance the true and false positives and negatives more effectively, leading to better overall predictive accuracy. This highlights the importance of the interplay between the inclusion and exclusion criteria and the prediction models for the overall model performance.

## Discussion

We explored the effectiveness of 18 different language models in streamlining the study selection process for three SRs, particularly in title and abstract screening. The reduction in workload for one reviewer from reading all identified articles to only those which could not be classified and those with conflicting recommendations on inclusion and exclusion between the first reviewer and the LLM was on average 92% (368 instead of 4,662), 88% (202 instead of 1741), and 40% (40 instead of 66). Working on the inclusion and exclusion criteria improved the performance of the models, highlighting the importance of the interplay between the model and the defined inclusion and exclusion criteria for the model’s performance.

## Methodological Discussion

### Used Reviews.

The three utilized reviews were chosen based on their different topics and methodological quality. Reviews I followed the PRISMA guidelines, and Review II (Neuro) mentions adherence to the Cochrane Collaboration methodology. Review III (DigiHealth) does not specify any recommendations in its methodology. While the former two states follow recommendations, they still do not adhere completely and have some limitations, like missing information on the date when the search was performed. Nonetheless, considering the overall quality, the former two adhere to a greater extent to the current recommendations, which aim to secure the production of a high-quality review. Surprisingly, we observed that involving a LLM in the study selection of a general high-quality review result did not result in better performances compared to a review with lower quality. Contrary Review III (DigiHealth) had slightly better results. This might be the case since only one author screened titles and abstracts, and only a few studies were included in Review III due to rather narrow search terms. Overall, setting up a LLM and validating the inclusion and exclusion criteria for a narrow review like this might take as much time as screening the identified articles of that search; thus, applying an LLM to few identified articles will likely not reduce the workload.

### Data Preprocessing.

One challenge with the use of LLMs as a reviewer to screen the title and abstracts arises during the data preprocessing and when the model tries to classify a record. During these processes, information for some of the originally identified articles is not retrieved completely. This means that the input of the LLM lacks information—in some cases, the whole abstract or title. In our approach, those articles would surely not pass the LLM-assessed criteria, meaning the LLM returns false for all criteria, which might lead to record exclusion. However, if this article is included by the first reviewer, it would need to be discussed or checked by an additional reviewer regardless. Thus, the exclusion of the record by the LLM does not threaten the review’s validity but requires either some extra effort to solve the problem or a slightly increased workload for the additional reviewer, whose workload ought to be significantly reduced by the LLM.

### Inclusion and Exclusion Criteria.

If the inclusion and exclusion criteria are well defined, the LLMs perform better in the study selection. Though this also applies to humans, consequently, with our work, we confirm the importance of setting and defining the inclusion and exclusion criteria thoroughly. The inclusion of at least two human reviewers is important. If only one person defines the inclusion and exclusion criteria and performs the study selection process, the results are likely biased toward this person. Therefore, a second reviewer should also be included in setting the inclusion and exclusion criteria, independent of utilizing a LLM for study selection or another human, to prevent biasing results. Our additional analyses prove that it is possible to optimize the inclusion and exclusion criteria based on the model’s outputs for the individual inclusion and exclusion criteria. Through this optimization, we can improve the accuracy and effectiveness of SRs while also minimizing manual sifting. However, how the inclusion and exclusion criteria are best refined, besides including a second person, is beyond the scope of this publication.

Moreover, another significant challenge we encountered was the occasional failure of the LLM to produce correctly formatted outputs, which subsequently hindered our ability to parse the data effectively. For instance, some outputs were prefaced with unnecessary text, such as “Here is the output in the requested format”, which led to parsing errors. This extraneous text caused the system to fail in recognizing and processing the content properly, resulting in the inability to analyze specific records. Such instances illustrate a critical issue where the LLM does not strictly adhere to the instructions to return only formatted information, affecting the overall analysis. This explains why, in some combinations of SR datasets and LLMs, we were unable to process all records effectively. Ensuring that the LLM consistently generates outputs in the exact required format is essential for the reliability and efficiency of our system in handling SRs.

### Decision on the Final Inclusion.

The SR process benefits significantly from high recall, ensuring that no relevant studies are missed and thus maintaining the review’s comprehensiveness. However, when applying LLMs, this should be balanced against precision to avoid an overload of irrelevant articles, making the review process more efficient. The F1-score is a helpful metric in this regard, as it balances precision and recall. A relatively low F1-score suggests that there is room for improvement in optimizing this balance.

When selecting articles based on the condition that all criteria must be true, we found that this approach was highly stringent. This method resulted in a limited selection of articles, as any single false criterion would exclude an article from being selected. The confusion matrices and aggregated metrics demonstrate that the models’ performance, particularly in terms of accuracy, recall, and F1-score, was rather poor and varied significantly between reviews. The strictness of this approach led to a higher number of false negatives, as many articles that could have been relevant were excluded due to not meeting one or more criteria.

In contrast, our supplementary analysis approach using an RF classifier allowed for a more nuanced and flexible selection of articles. By leveraging the predicted Boolean values for each criterion, the RF model could account for the complex interplay between different criteria and predict the screening 1 outcome more effectively.

The RF approach’s flexibility is particularly advantageous because it does not require all criteria to be true simultaneously. Instead, it considers the relative importance of each criterion and how they collectively contribute to the likelihood of an article meeting the screening 1 criteria. This method reduces the risk of excluding potentially relevant articles due to minor deviations in one or more criteria, thus improving the inclusiveness and accuracy of the selection process.

Overall, the inclusion of this approach highlights the limitations of strictly satisfying all criteria as a selection method and demonstrates the importance of working on the inclusion and exclusion criteria prior to applying any LLM. The RF model’s ability to integrate and weigh multiple criteria results in more reliable and accurate predictions, ultimately enhancing the efficiency and effectiveness of the initial screening process in SRs.

### Interpretation of the Results.

The pattern of missing records can be attributed to several key factors related to dataset characteristics and LLM processing capabilities. The larger size of Review I (Physio, n = 4,501), increased the likelihood of encountering edge cases—records with unusual formatting, complex content structures, or characteristics that challenge LLM processing limits. The consistently small number of missing records (1–6) across different models suggests these might be specific “difficult” records sharing challenging characteristics rather than random processing failures. This hypothesis is supported by the perfect completion rates achieved in the smaller Reviews II and III, which were less likely to contain such edge cases and posed lower processing overhead. These findings highlight the importance of robust error-handling mechanisms in SR automation systems, particularly when processing large-scale reviews.

Evaluating models using MCC and PABAK provides a more nuanced perspective on performance compared to traditional metrics such as accuracy. MCC incorporates all four elements of the confusion matrix, making it a dependable indicator of model quality, particularly in cases of class imbalance. PABAK, on the other hand, assesses the agreement between model predictions and ground truth, offering valuable insights into the reliability of model outputs. Together, these metrics provide a well-rounded evaluation framework, crucial for identifying models that excel in various aspects of classification tasks.

Investigating the difference in performance between the LLMs, we see variations for the workload reduction for a second reviewer by 89% to 93%, 83% to 90%, and 33% to 56% for the three reviews, respectively. In our additional analyses, we found that the remaining performance of all models could be improved by modifying the inclusion criteria. Hence, we recommend that prior to the utilization of a LLM, the inclusion and exclusion criteria be modified with the help of the LLM. This could, for example, include a training set of records with certainly included and excluded articles. However, investigating the ideal opportunity to improve the inclusion and exclusion criteria of a review is beyond the scope of this article.

It should be noted that the inclusion of a second researcher in the screening process normally yields a 6.6 to 9.1% increase in articles (14, 15). Furthermore, the Cohen kappa of two reviewers during the full text screening is often around 0.8 (16). The articles on which the reviews do not agree regarding inclusion and exclusion are either discussed between the reviewers or an additional expert is consulted. Thus, the current standard of including two researchers does not guarantee flawlessness. Consequently, requesting a 100% overlap in the included and excluded studies with the LLM for screening 1 would be too conservative. We found that, as in two humans, the decision to include or exclude articles deviates between the LLM and the researchers. In the study selection process, relevant articles might still be excluded. However, if we stick to the assumption that humans are the standard, a reduction in the title abstract screening workload for one of the involved reviewers between 33% and 93% is achievable. However, the participation of an LLM adds some more effort because the LLMs must be set up, but we are now developing user-friendly solutions to alleviate this strain. Finally, refining the inclusion and exclusion criteria with an LLM prior to application can reduce the workload even further and is likely to improve the quality, as indicated in our supplementary analyses.

To sum up, our results showcase the distinctive reasoning abilities of the models, not only facilitating the rapid scanning of large volumes of articles but also providing insights that may assist researchers in refining their inclusion and exclusion criteria. Employing this methodology, reminiscent of approaches used by human researchers, may enhance the accuracy of SRs. Irrespective of the ongoing debate on speed, the efficiency of LLMs and human judgment streamline the review process, underscoring the potential of an LLM companion to augment human efforts in SRs.

### Future Directions.

As an increasing number of research articles surface annually, the significance of SRs is on the rise. Conducting a high-quality SR is a time-consuming and resource-intensive process. Future LLMs will possess the capability to enhance and expedite this process at three distinct stages: 1) defining and particularly identifying synonyms for search terms; 2) selecting studies through title, abstract, and full-text screening; and 3) aiding in data extraction.

This manuscript offers initial insights into the performance of LLMs in comparison to the current gold standard—humans—specifically in selecting relevant studies based on titles and abstracts. The inclusion of 18 different LLMs highlights that any (of the selected) LLM had the potential to reduce the workload for a second reviewer, though to a different extent. We therefore assume that also upcoming LLMs will perform similarly or, with further development, maybe even better than our findings indicate, though validation may be required. Further, our findings underscore that the precise specification of inclusion and exclusion criteria is pivotal for selecting appropriate records for LLMs. Given that these criteria also inform the generation of search terms, we posit that to fully harness the potential of LLMs in the review process, they should be involved throughout the entire process.

The future role of a LLM can be likened to that of a second researcher in the SR process. Attention is warranted for suggestions on synonyms as well as discrepancies in included and excluded articles or extracted data. Yet, a LLM does not replace a researcher completely, but it reduces the workload and serves as an additional knowledge source, albeit with potential limitations.

## Conclusion

LLMs are a powerful tool to reduce labor in time-consuming SRs for one reviewer by 33 to 93%. The model’s performance depends on the interplay between the inclusion and exclusion criteria and the models. Refinding the inclusion and exclusion criteria has the potential to further reduce the workload of a reviewer. Thus, to harness the full potential of LLMs in the SR process, we suggest utilizing them early on in the process. However, verifying research for the utilization of LLMs throughout the full process is still wanted.

## Methods

We first give a brief summary of the SRs, whose “study inclusion results” were used in this benchmarking study. We aim to contrast the selection approach with that of an LLMs, which will be elaborated upon in the subsequent methodological segment. In the final sections, we will explain how we compare the results between the existing review and the LLM.

### Summary of Systematic Reviews Utilized.

An overview of the included studies for 1) title and abstract screening, 2) full text screening, and 3) final inclusion for all three reviews is provided above in Table 1.

### Review I - Economic Evaluations of Musculo-Skeletal Physiotherapy (Physio).

The utilized SR provides an overview of existing full economic evaluations in the field of musculo-skeletal physiotherapy (17, 18), which adhere to the PRISMA guidelines. We utilized the systematic search of the review, which was conducted in 2022 (not including the final updated search), in three databases: Medline (through PubMed), EconLit, and NHS-EED (which can only be searched up to March 2015). “Economic evaluation”, “physiotherapy”, and “musculoskeletal” were the main search terms used together with their acronyms, and they were combined with “AND”. Details on the specific searches and terms can be found elsewhere (17). Additionally, included studies in relevant SRs were identified by researchers, and references to the included articles were searched for additional relevant articles. This combined search strategy resulted in 4,662 articles without duplicates.

The following study selection was performed by two independent researchers. They applied the original, predefined inclusion and exclusion criteria (Table 2) in a two-step process. First, they screened the title and abstracts. This led to the exclusion of 4,481 articles and the inclusion of 185 articles for the subsequent step of full-text screening. Finally, 75 articles were included.

**Table 2.** Criteria used in screening 1 of Review I (Physio)

Specific Criteria	Description
Population	If the study population comprises patients with musculoskeletal conditions, with no majority having another primary disease or intellectual disabilities, then return true. Otherwise, return false.
Intervention	If the treatment involves physiotherapy (techniques like exercises, manual therapy, education, and modalities such as heat, cold, ultrasound, and electrical stimulation to aid in patient recovery, pain reduction, mobility enhancement, and injury prevention), or at least one of the intervention/control group treatments was provided exclusively by physiotherapists, then return true. However, if the treatment of interest was offered by an interdisciplinary team, non–health care professionals, or mostly by a different profession to physiotherapists, then return false.
Physiotherapy and another treatment	In case at least one of the intervention/control group treatments was provided exclusively by physiotherapists, if the intervention includes physiotherapy and another treatment and the other treatment is provided in a comparator group, then return true.
E-interventions	If the study evaluates the economic aspects of E-interventions, digital interventions or eHealth interventions, then return false. Otherwise, return true.
Control Group	If there is a control group of any type—for example, wait and see, usual care, placebo, or alternative treatments, then return true. Otherwise, return false.
Outcome	If the outcome of the study involves or allows a full economic evaluation, potentially including cost-effectiveness ratios and cost-utility ratios or if the study provides information on the costs and clinical effects of a treatment then return true. Otherwise, return false.
Study type	If the article is not a conference abstract, review, study without results (like a protocol), or model-based study, then return true. Otherwise, return false.

### Review II: Treatment Effectiveness in Hereditary Peripheral Neuropathies (Neuro).

The SR presented in ref. 19 focuses on the effectiveness of pharmacological and gene-based treatments for hereditary peripheral neuropathies. Its design follows the Cochrane Collaboration methodology (20). The review employed a comprehensive search across multiple databases, including the Cochrane Central Register of Controlled Trials (CENTRAL), the Cochrane Neuromuscular Disease Group Specialized Register, ClinicalTrials.gov, the European Clinical Trials Database (EudraCT), the WHO International Clinical Trials Registry (ICTRP), and the Centre for Reviews and Dissemination (CRD), as well as PubMed for an expanded search. The two main search terms were “neuropathy” and “inherited”. Besides these databases, the authors evaluated the references to identify additional relevant articles. Initially, 1,898 articles were identified for screening.

After the removal of 157 duplicates, 1,741 titles and abstracts were screened by the researchers to evaluate whether they met the predefined inclusion criteria (Table 3). This initial screening step was passed by 119 articles. At this stage, 3 additional articles, identified through other sources, were added, bringing the total to 122 articles. After screening the full texts, 34 articles were included in the final analysis.

**Table 3.** Criteria used in screening 1 of Review II (Neuro)

Specific criteria	Description
Disease	If the condition studied was hereditary peripheral neuropathy confirmed genetically with known neuropathy genes or by pedigree where a significant proportion is genetically confirmed, then return true. Otherwise, return false.
Treatment	If the study focuses on pharmacological therapies or genotype-related dietary changes specifically targeting the neuropathy, return true. Nonpharmacological interventions such as physiotherapy, surgery, and genetic counseling should be excluded, return false.
Human	If the study involves human participants, return true. Otherwise, return false.
Genetic	If the study involves patients who are genetically confirmed to have mutations in known neuropathy genes, or are familial relatives of someone who was genetically diagnosed, or have been diagnosed through accepted pathological diagnostic criteria are strongly linked to genetic variants (such as TTR amyloidosis), then return true. Studies including patients diagnosed only on a symptomatic basis without genetic confirmation or acceptable pathological criteria are excluded, and returned as false.
Results	If the study reports results from interventions that were explored in databases such as ClinicalTrials.gov, CENTRAL, EudraCT, ICTRP, and CRD with filters ensuring only interventional studies with results available were included, then return true. Additionally, studies should present results for genetically confirmed cohorts separately if the cohort includes both diagnosed and undiagnosed patients. Otherwise, return false.

### Review III: Cost-Effectiveness of AI in Healthcare (DigiHealth).

The SR presented in ref. 21 was designed to evaluate the cost-effectiveness of AI applications within healthcare settings and to assess whether these studies adhere to predefined quality criteria, which were based on traditional and adapted frameworks used for assessing cost impacts.

A systematic literature search identifying only articles published in English during the prior 5 y was conducted in the PubMed database only. The two main search terms “AI” and “cost effectiveness” were supplemented with one and two synonyms in the searches, respectively. In total, 66 articles were identified. The inclusion criteria (Table 4) were particularly focused on identifying studies that provided a thorough economic evaluation of AI technologies in healthcare settings. The title and abstract screening steps were performed by one researcher, and 45 articles passed this initial step. Ultimately, after full text screening, 6 articles were included in the review.

**Table 4.** Criteria used in screening 1 of Review III (DigiHealth)

Specific criteria	Description
AI Functionality Description	Return true if the study provides a comprehensive description of an AI functionality used in healthcare; otherwise, return false.
Economic Evaluation	Return true if the study evaluates the economic efficiency and outcomes of an AI application in healthcare, specifically assessing cost-effectiveness or return on investment; otherwise, return false.
Quantitative Healthcare Outcomes	Return true if the study reports quantitative outcomes in at least one healthcare system, showing measurable impacts such as patient recovery times, treatment efficacy, or cost savings; otherwise, return false.
Relevance to AI in Healthcare	Return false if the title of the study does not explicitly cover a topic related to AI in healthcare, indicating the study is not primarily focused on AI applications within healthcare; otherwise, return true.
AI Application Description	Return false if the abstract does not contain a description of an AI application in healthcare, indicating a lack of focus on how AI technologies are implemented or their functional roles within healthcare; otherwise, return true.
Economic Outcome Details	Return false if the abstract or full text does not elaborate on the quantitative economic outcomes in one healthcare system, failing to provide specific economic data or analysis related to the AI application; otherwise, return true.

### Article Data Preprocessing.

For all three reviews, we received the articles in tabular data from the authors of the original reviews, where each row represents an article or record. For each row, we obtained multiple columns, including “title” and “abstract”, as required by the first screening of the PRISMA SR process. Additionally, we obtained the column screening 1, which indicates whether studies were included or not after title and abstract screening according to human reviewers. Although not directly relevant for our implementation, we also obtained a column screening 2, which indicates whether studies were included or not after full text screening according to human reviewers.

In all cases, the Title and Abstract columns were joined together to create a new column called “Record”. Rows where the Abstract column was empty were also removed, ensuring that only entries with valid abstracts were retained for analysis. We excluded duplicate records and those not available in English. Finally, all records were assigned a unique article identifier that would be used for later LLM screening. The summary of records that serve as the starting point for our analysis after data preprocessing is shown in Table 1.

### Data Embedding.

For each SR, we implemented a straightforward document embedding approach using the Chroma vector database. The process began by loading complete articles from the preprocessed datasets, with each article’s full text from the Record column treated as a single unit. Each document was assigned a unique identifier to maintain consistency throughout the screening process.

The embedding process utilized either OpenAI’s or local embedding models, depending on the specific implementation. We selected the newly created column ‘Record’ to be embedded, using different embedding techniques based on the models employed. When working with OpenAI models, we utilized OpenAI’s text-embedding-3-large embedding models to transform the text data into vector representations (22). For open-source LLMs, we employed the Nomic-Embed-Text model (23), which was locally deployed using Ollama (24). These embeddings were stored in a Chroma vector database (25). For efficient processing, documents were embedded in batches of 100, with both the full text content and associated metadata added to the Chroma database.

We implemented the Chroma vector database primarily for its powerful filtering capabilities during retrieval operations. Specifically, Chroma allows us to filter the vector store by unique identifiers each time we query the database, ensuring that only the relevant Record (title and abstract) is retrieved for each screening decision. This filtering functionality is crucial for our screening pipeline as it guarantees that the LLM evaluates the correct article every time, eliminating potential cross-contamination between documents during the screening process. The choice of Chroma over other vector stores was driven by this built-in filtering feature, which enables precise document retrieval and maintains the integrity of our screening decisions. Additionally, Chroma provides robust error handling and atomic save operations, further ensuring data reliability throughout the screening process.

To ensure data integrity, we implemented comprehensive validation checks. The system verified that all documents were correctly embedded by comparing the unique identifiers in the original dataset against those in the Chroma index. This validation process identified any missing or extra documents, with warnings generated for discrepancies. The embedding validation was particularly crucial for maintaining consistency across the three SRs, which contained 4,501, 1,650, and 66 articles, respectively.

### Inclusion/Exclusion Preprocessing.

Even though our LLM decided for each individual criterion if it was met or not, a record was finally only included when all specific criteria were true. The respected specific inclusion and exclusion criteria from the reviews as utilized by the LLMs may be found in Tables 2–4.

### Study Selection Approach with the LLMs.

For the inclusion/exclusion process, we developed a structured screening pipeline using ChatPromptTemplate that standardizes the evaluation of articles against the predefined screening criteria. The prompt template explicitly instructs the LLM to analyze the provided scientific article and determine whether it meets the specified criteria, with each criterion requiring a Boolean response (True/False) and a brief justification. The LLM’s responses are structured as JSON objects to ensure consistent and parseable outputs.

Our implementation utilizes a Chroma vector database to manage document processing. For each article, we leverage Chroma’s filtering capabilities to retrieve the specific document using its unique identifier, ensuring that exactly the correct article is processed during each screening decision. This filtering mechanism is crucial for maintaining the integrity of our screening process, as it guarantees that the LLM evaluates precisely the intended document each time.

The screening pipeline includes robust error handling and data management mechanisms. We implemented atomic save operations to prevent data corruption and maintain separate dictionaries for successfully processed articles and those that encountered processing issues. The system validates the mutual exclusivity of these sets to ensure data integrity. Progress is automatically saved after processing each batch of articles, enabling the analysis to be resumed if interrupted.

For concurrent processing, we implemented an asynchronous execution framework with a controlled concurrency limit (five simultaneous processes) to manage system resources effectively. The system includes comprehensive logging and validation checks, tracking successful and failed processing attempts. When processing errors occur, the system distinguishes between rate-limit-related errors and other types of failures. For rate-limit errors, the system implements an exponential backoff strategy, waiting progressively longer between retries (2^attempt seconds) for up to five attempts per document. Non-rate-limit errors are logged and the document is moved to a separate tracking set of missing records. This approach ensures both efficient processing and comprehensive error handling while maintaining system stability under various failure conditions. To ensure reliable outputs, we implemented a JSON response parser that handles various edge cases and standardizes the Boolean values across all criteria. The parser validates both the structure and content of LLM responses, converting string representations of Boolean values to proper Boolean types and providing appropriate default values when necessary.

The LLMs tested in our work are presented in Table 5. Open-source models were locally deployed using Ollama (24). We utilized the LangChain framework to provide a unified deployment for all models. For OpenAI models (gpt-3.5-turbo-0125, gpt-4o-mini-2024-07-18, gpt-4o-2024-05-13), we used LangChain’s ChatOpenAI wrapper with temperature set to 0.0, top_*P* = 0.95, and a fixed random seed of 28 for reproducibility. For open-source models, we employed LangChain’s ChatOllama wrapper, accessed through a secure HTTPS endpoint, with several crucial parameters: temperature set to 0.0, a context window of 25,000 tokens to accommodate models with varying context sizes, top_*P* = 0.95, and num_predict set to −1 to allow unlimited response length needed for complete JSON outputs. The context window and unlimited response length settings were particularly important as they ensured all models could process the same amount of input text and generate complete screening decisions with justifications, regardless of their underlying architecture. All models were configured to output responses in JSON format, ensuring consistent interaction patterns and response handling across the entire evaluation.

**Table 5.** Summary of LLMs and embedding models used upon analysis

LLM name	Model family	Parameters	Quantization	Provider	Embedding model
gpt-3.5-turbo-0125	GPT	Unknown	Unknown	OpenAI	text-embedding-3-large
gpt-4o-mini-2024-07-18	GPT	Unknown	Unknown	OpenAI	text-embedding-3-large
gpt-4o-2024-05-13	GPT	Unknown	Unknown	OpenAI	text-embedding-3-large
gemma:7b	Gemma	9B	Q4_0	Google	nomic-embed-text
gemma2:9b	Gemma	9.2B	Q4_0	Google	nomic-embed-text
gemma2:27b	Gemma	27.2B	Q4_0	Google	nomic-embed-text
llama3:8b-instruct-fp16	Llama	8.0B	F16	Meta	nomic-embed-text
llama3:8b	Llama	8.0B	Q4_0	Meta	nomic-embed-text
llama3.1:8b	Llama	8.0B	Q4_0	Meta	nomic-embed-text
llama3.1:70b	Llama	70.6B	Q4_0	Meta	nomic-embed-text
llama3.2:3b	Llama	3.21B	Q4_K_M	Meta	nomic-embed-text
reflection:70b	Llama	70.6B	Q4_K_M	Reflection AI	nomic-embed-text
llama3-Athene-70b	Llama	70.6B	Q4_0	Nexusflow Team	nomic-embed-text
mistral:v0.2	Llama	7B	Q4_0	Mistral AI	nomic-embed-text
mistral-nemo:12b	Llama	12.2B	Q4_0	Mistral AI	nomic-embed-text
mistral:7b (v0.3)	Llama	7B	Q4_0	Mistral AI	nomic-embed-text
mixtral:8x22b	Llama	140.6B	Q4_0	Mistral AI	nomic-embed-text
qwen2.5:7b	Qwen2	7.26B	Q4_K_M	Qwen Team	nomic-embed-text

Models include proprietary GPT variants from OpenAI and open-source options like Gemma, Llama, and Mistral. “Parameters” shows model size in billions, where known. “Q4_0” indicates 4-bit quantization (26), “F16” is 16-bit floating-point (27). OpenAI models use “text-embedding-3-large” for embeddings, while open-source models use “nomic-embed-text.”

### Comparison between Selected Studies of Human Reviewers and our LLMs.

In our study, we evaluate the effectiveness of LLMs in screening titles and abstracts for SRs, comparing their performance against that of human reviewers. To conduct this analysis, we employ datasets containing literature reviews, of which we know the number of included articles after screening 1.

In a first step, we investigated the potential workload reduction by utilizing an LLM as a second reviewer in the title and abstract screening process. Therefore, we added the number of articles that were classified as included by both the first human reviewer and the LLM to the number of articles that were classified as excluded by both the first reviewer and the LLM. We then calculated the mean number of these correctly classified articles among all LLMs per SR. Subsequently, we calculated the percentage of this mean number of correctly classified articles per review. For this calculation, we considered the original identified number of articles in the SRs—not the preprocessed number of included articles. Articles that were dropped during preprocessing would need to be checked by a second human reviewer, as would those articles with conflicting classifications between the first human reviewer and the LLM; therefore, they are part of the remaining workload for a second human reviewer. Additionally, we evaluated the minimum and maximum number of correctly classified articles per review and calculated their respective percentages.

For a deeper quantitative assessment of the model’s performance, we calculate a range of standard statistical metrics using the scikit-learn Python package (28). We generate a confusion matrix to provide a detailed comparison of true positives, false positives, true negatives, and false negatives between the LLM’s predictions and the judgments of human reviewers. Additionally, we compute precision to measure the accuracy of the LLM’s positive predictions and recall to assess its ability to capture all relevant instances. The F1 score is calculated to provide a balance between precision and recall, which is particularly useful in scenarios where the class distribution might be uneven (29). Furthermore, we compute the MCC to offer a balanced measure of the binary classifications’ quality, suitable even when class sizes are disparate, as is often the case in SRs.

To assess the agreement between two raters on a classification task, we calculated both Cohen’s kappa and the prevalence-adjusted bias-adjusted kappa (PABAK). Unlike typical interrater reliability metrics, which estimate chance agreement using marginal probabilities, PABAK reduces this to a constant 0.5, making it especially useful when one category dominates or raters’ categorization thresholds disagree. To compute PABAK, we first determined the observed agreement (p_0_), the proportion of instances where both raters concurred. The PABAK score was then calculated using the formula (PABAK = 2p_0_ − 1), scaling the observed agreement to adjust for chance, yielding values from −1 (perfect disagreement beyond chance) to +1 (perfect agreement beyond chance), with 0 indicating agreement exactly as expected by chance. This method provides a robust measure of agreement, corrected for any biases and prevalence effects.

### Additional Analyses.

To understand the relationships between the Boolean criteria predicted by LLMs and the target variable (screening 1), we calculated the correlation matrix using Pearson’s correlation coefficient. This analysis helped in identifying which features were strongly correlated with the screening 1 column, thus providing insights into the predictive power of each feature. The correlation matrix was visualized using heatmaps to highlight significant correlations.

In supplementary analyses, we evaluated an alternative approach to decision-making using RF classification. Instead of requiring all criteria to be true for inclusion, we trained RF classifiers using the LLM-predicted criteria as features to predict the final screening decision (30). The idea that it might be unduly restrictive to require all Boolean criteria to be true served as the basis for this methodological change. To test this, we used the LLM-predicted Boolean values for each review, aiming to demonstrate their predictive power for the screening 1 outcome.

The RF classifiers were implemented using scikit-learn with 100 estimators and balanced class weights to address the inherent class imbalance in SRs. For cross-validation, we implemented an adaptive approach based on dataset size: While we aimed for fivefold cross-validation, the system automatically adjusted the number of folds (minimum 2, maximum 5) based on the minimum class size to ensure reliable validation even with smaller datasets. For example, in Review III (DigiHealth) with only 66 samples, this adaptive approach ensured sufficient samples in each fold for both positive and negative classes.

To maintain consistency in evaluation, we computed the same comprehensive set of metrics as used in the all-criteria-true approach: precision, recall, specificity, F1-score, MCC, Cohen’s kappa, and PABAK. The predictions were generated using cross_val_predict to ensure unbiased evaluation, and the results were tracked through a progress bar that displayed real-time counts of successful predictions. This parallel implementation of both approaches (all-criteria-true and RF classification) enabled direct comparison of their effectiveness in screening decisions.

## Supplementary Material

Appendix 01 (PDF)

## Data Availability

The code data have been deposited in Github (https://github.com/fmdelgado/LLM_SR_medicine/) (31). All other data are included in the manuscript and/or *SI Appendix*.
