# Supplementary material for: Transforming literature screening: The emerging role of large language models in systematic reviews
Source: Proc Natl Acad Sci U S A. 2025 Jan 6;122(2):e2411962122. doi: 10.1073/pnas.2411962122 (PMC11745399; doi:10.1073/pnas.2411962122)
Supplement: Supplementary file 1 — Appendix 01 (PDF) [file pnas.2411962122.sapp.pdf]

PNAS

Supporting Information for
Transforming Literature Screening: The Emerging Role of Large Language Models in Systematic Reviews

Authors: Fernando M. Delgado-Chaves[1], Matthew J. Jennings[2], Antonio Atalaia[3], Justus Wolff[4], Rita Horvath[5], Zeinab M. Mamdouh[6,7], Jan Baumbach[1,8], Linda Baumbach[9,10]

[1]Institute for Computational Systems Biology, University of Hamburg, Germany

[2]Center for Motor Neuron Biology and Diseases, Department of Neurology Columbia University, New York, NY 10032

[3]Inserm Center of Research in Myology, Neuro-Myology Service G.H. Pitié-Salpêtrière, Sorbonne Université, Paris, France

[4] Syte Strategy Institute, Hohe Bleichen 8, 20354 Hamburg

[5]Department of Clinical Neurosciences, University of Cambridge, Cambridge, UK

[6]Department of Pharmacology and Personalised Medicine, Maastricht University, The Netherlands

[7] Department of Pharmacology and Toxicology, Zagazig University, Egypt

[8]Institute for Mathematics and Computer Science, University of Southern Denmark, Denmark

[9]Department of Health Economics and Health Services Research, University Medical Center Hamburg-Eppendorf, Germany

[10]Center for Bioinformatics Hamburg, MIN-Faculty, Universität Hamburg, Hamburg, Germany

Paste Fernando Miguel Delgado-Chaves
Email: fernando.miguel.delgado-chaves@uni-hamburg.de

This PDF file includes:

Supporting text
Figures S1 to S8

Additional Confusion Matrices

These confusion matrices provide a detailed breakdown of classification outcomes (True Positive, False Positive, True Negative, and False Negative) for each systematic review. The x-axis represents the predictions made by the LLMs, while the y-axis shows the human reviewer classifications. Darker shades in the matrices indicate higher frequencies of classifications in each quadrant.

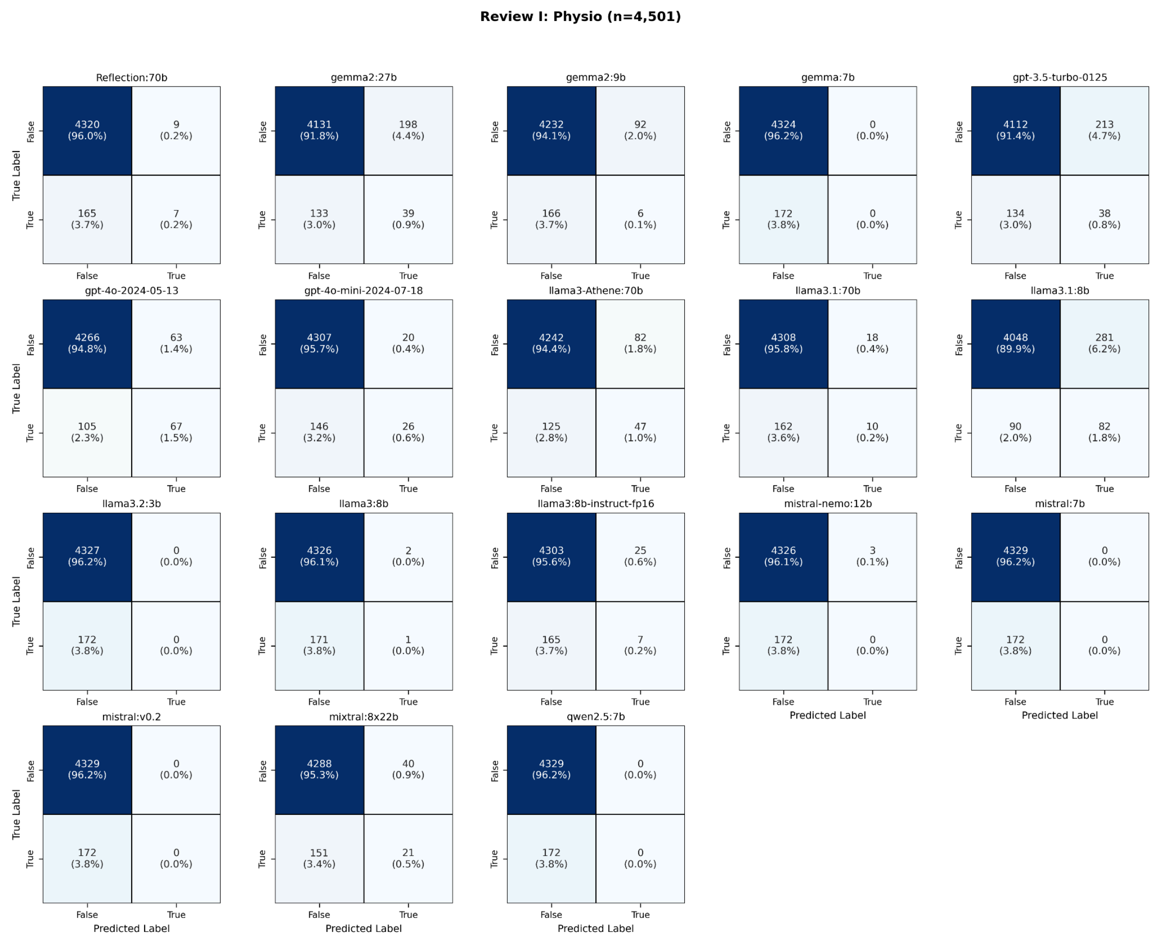

Fig. S1. Confusion Matrices for Models in Review I (Physio) , when an article is predicted as selected (True) if all criteria are True. Each subplot represents the confusion matrix of a different model in a given review. The confusion matrices depict the true negative (TN), false positive (FP), false negative (FN), and true positive (TP) classifications of binary labels. The x-axis represents the predicted labels, while the y-axis shows the actual labels. The annotations provide the count of predictions for each quadrant, with the total number of records for each model mentioned in the x-axis label. The title of each subplot indicates the LLM name. The matrix color intensities indicate the frequency of predictions, with darker colors representing higher counts.

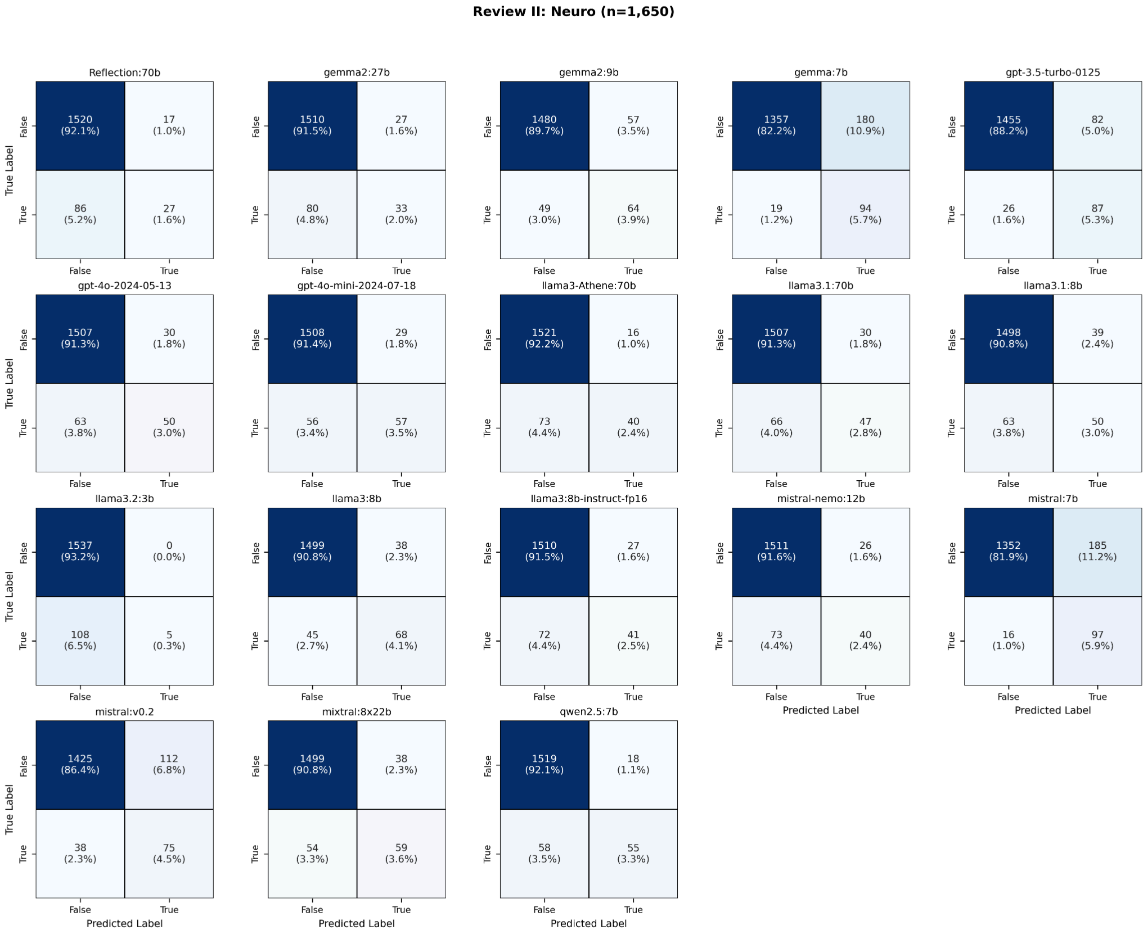

Fig. S2. Confusion Matrices for Models in Review II (Neuro) , when an article is predicted as selected (True) if all criteria are True. Each subplot represents the confusion matrix of a different model in a given review. The confusion matrices depict the true negative (TN), false positive (FP), false negative (FN), and true positive (TP) classifications of binary labels. The x-axis represents the predicted labels, while the y-axis shows the actual labels. The annotations provide the count of predictions for each quadrant, with the total number of records for each model mentioned in the x-axis label. The title of each subplot indicates the LLM name. The matrix color intensities indicate the frequency of predictions, with darker colors representing higher counts.

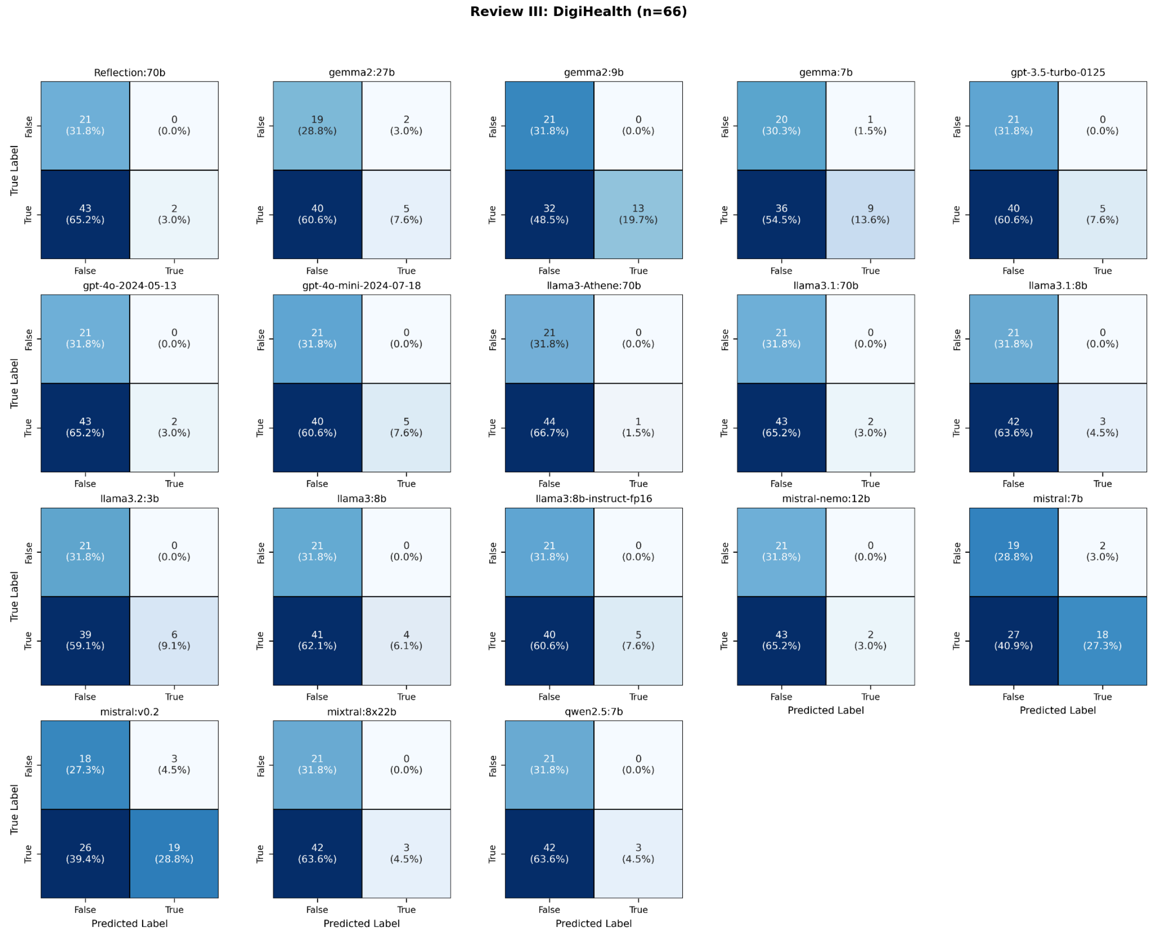

Fig. S3. Confusion Matrices for Models in Review III (DigiHealth) , when an article is predicted as selected (True) if all criteria are True. Each subplot represents the confusion matrix of a different model in a given review. The confusion matrices depict the true negative (TN), false positive (FP), false negative (FN), and true positive (TP) classifications of binary labels. The x-axis represents the predicted labels, while the y-axis shows the actual labels. The annotations provide the count of predictions for each quadrant, with the total number of records for each model mentioned in the x-axis label. The title of each subplot indicates the LLM name. The matrix color intensities indicate the frequency of predictions, with darker colors representing higher counts.

Random Forest Classifier as an Alternative for Article Selection

We evaluated an alternative approach to article screening using Random Forest (RF) classification, hypothesizing that requiring all criteria to be true might be overly restrictive. For each systematic review, we implemented RF classifiers using scikit-learn, configuring them with 100 estimators, a fixed random seed of 42 for reproducibility, and balanced class weights to handle the inherent class imbalance in systematic reviews. The features consisted of the Boolean criteria predicted by each LLM, with the human-determined "screening 1" decisions serving as ground truth.

Our preprocessing pipeline involved removing any records with missing values and converting all Boolean predictions to integer format (0 or 1). We implemented an adaptive cross-validation strategy based on dataset characteristics. For Reviews I and II, containing 4,501 and 1,650 articles respectively, we employed standard 5-fold cross-validation. For Review III (DigiHealth) , with only 66 articles, we adjusted the number of folds based on minimum class size to ensure reliable validation. This adaptive approach guaranteed sufficient samples in each fold for both positive and negative classes, particularly important for smaller datasets. To enable direct comparison with the all-criteria-true approach, we computed an identical set of performance metrics.

For each model and review combination, we maintained comprehensive tracking of processing metrics, including total number of processed samples, class distribution in both predictions and ground truth, and number of successful and failed processing attempts. The results were visualized through confusion matrices showing the distribution of predictions versus actual labels (Figures S4-S6), and performance metric comparisons between RF and all-criteria-true approaches across all models (Figure S7). This detailed analysis provided insights into the effectiveness of machine learning-based approaches for systematic review screening, offering an alternative to strict criteria-based selection while maintaining reliable screening performance.

The RF approach is generally beneficial, but it is not across all models. The results show clear trends but also highlight variability depending on the model used (Figure S8). Overall, the RF approach improved the MCC in around 75% of model-review combinations. This indicates that in most cases, the RF approach led to a higher MCC compared to the "all true" approach. The higher MCC suggests that RF is more effective in making accurate predictions in the majority of tested scenarios.

On a model-by-model basis, the benefits of the RF approach are uneven. For example, models like gpt-4o-2024-05-13 and gpt-4o-mini-2024-07-18 or llama consistently showed improvement across all reviews, indicating that RF effectively captured relationships in the data for these models. In contrast, models like gemma:7b showed only marginal or no improvements. This variability suggests that the effectiveness of RF depends on the specific model architecture and its training characteristics.

In terms of average MCC, the RF approach generally led to better performance compared to the "all true" approach. Boxplots confirmed that RF captures more nuanced data patterns, resulting in better classification performance. The "all true" approach, while simple, may be too rigid for diverse language models, limiting its adaptability to complex data patterns.

In summary, the RF pproach demonstrates that LLM-predicted criteria can be leveraged more effectively than the traditional "all true" requirement. While the "all true" approach requires all inclusion criteria to be met, RF can identify subtle patterns in how different criteria combinations predict inclusion, improving classification performance. However, the improvement varied across models and reviews, suggesting that preliminary performance assessment remains important when choosing between RF and "all true" approaches for specific systematic review tasks. This finding highlights the potential benefits of moving beyond rigid criterion-based selection toward more nuanced, machine learning-based screening strategies.

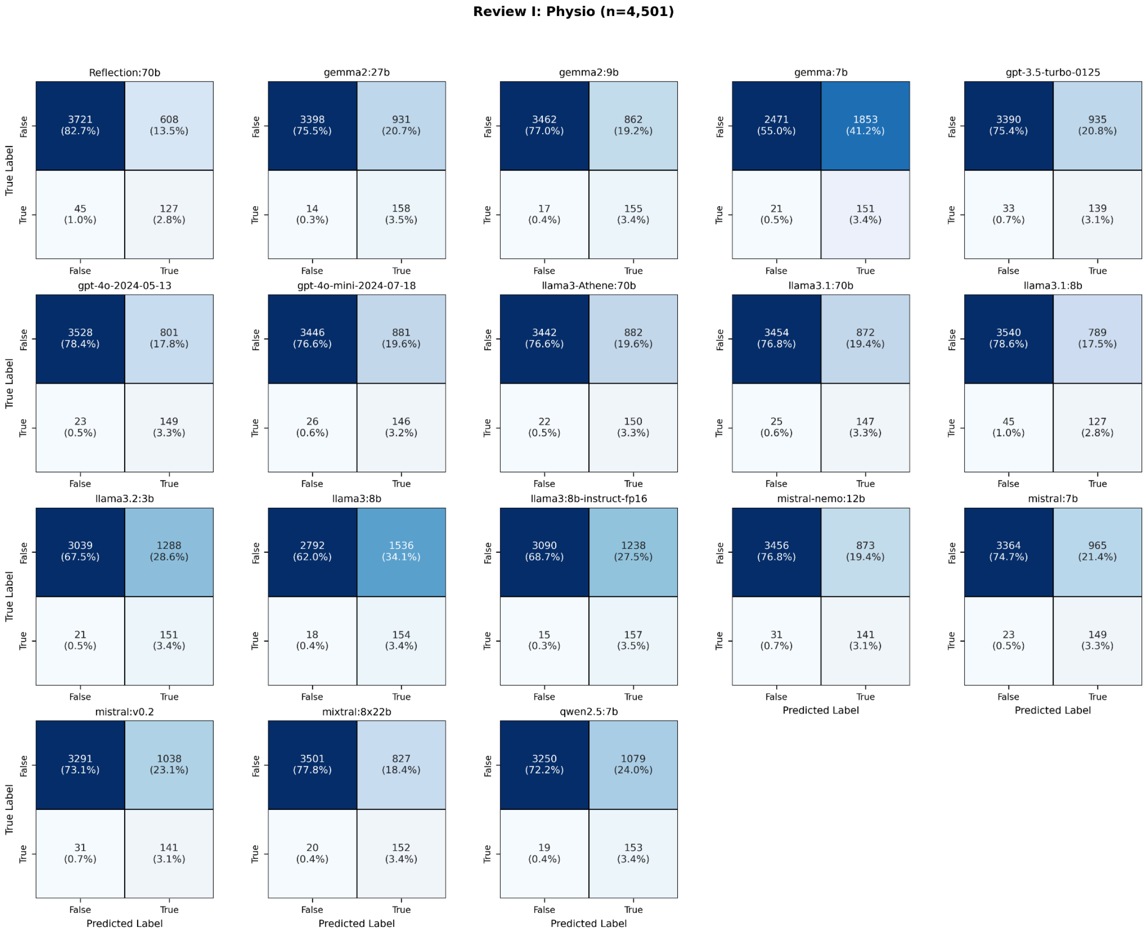

Fig. S4. Confusion Matrices for Models in Review I (Physio) , when an article is predicted as selected (True) using RF. Each subplot represents the confusion matrix of a different model in a given review. The confusion matrices depict the true negative (TN), false positive (FP), false negative (FN), and true positive (TP) classifications of binary labels. The x-axis represents the predicted labels, while the y-axis shows the actual labels. The annotations provide the count of predictions for each quadrant, with the total number of records for each model mentioned in the x-axis label. The title of each subplot indicates the LLM name. The matrix color intensities indicate the frequency of predictions, with darker colors representing higher counts.

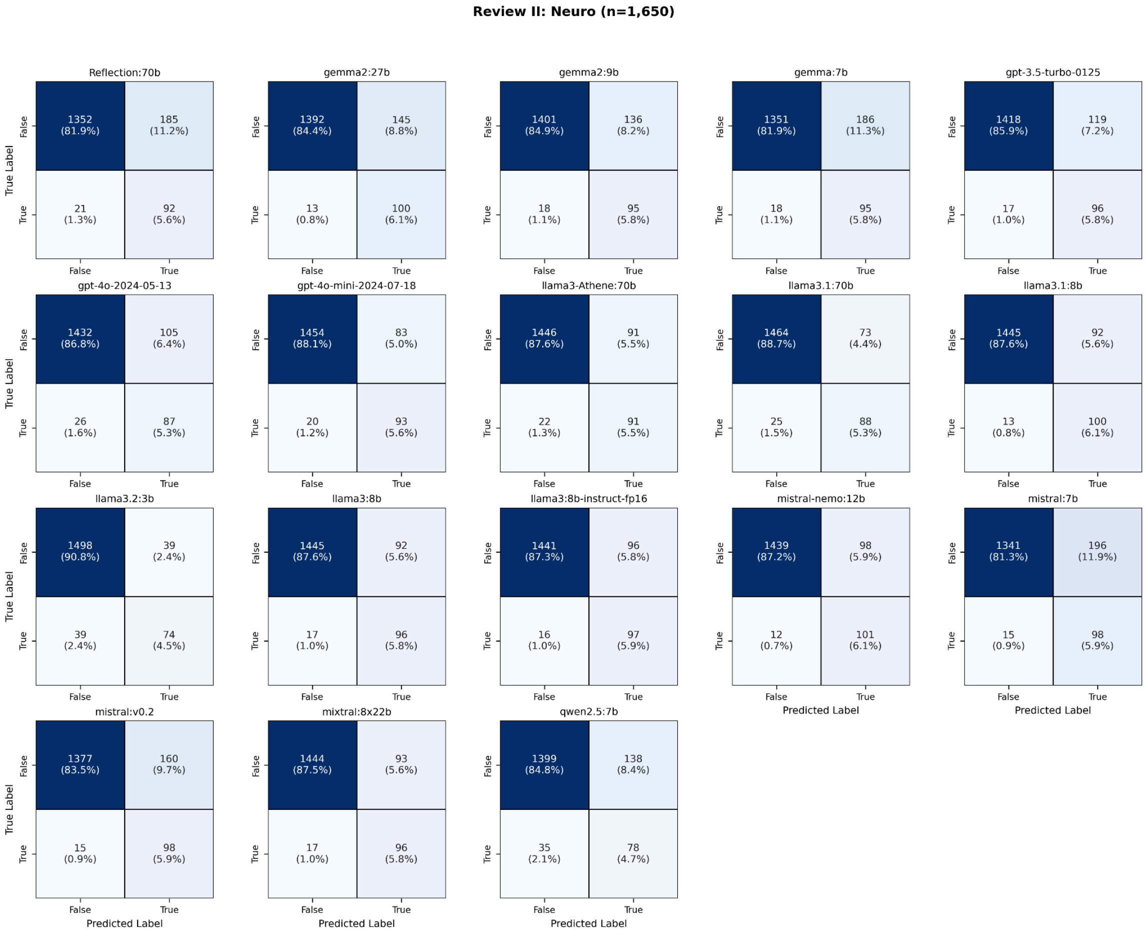

Fig. S5. Confusion Matrices for Models in Review I (Physio) , when an article is predicted as selected (True) using RF. Each subplot represents the confusion matrix of a different model in a given review. The confusion matrices depict the true negative (TN), false positive (FP), false negative (FN), and true positive (TP) classifications of binary labels. The x-axis represents the predicted labels, while the y-axis shows the actual labels. The annotations provide the count of predictions for each quadrant, with the total number of records for each model mentioned in the x-axis label. The title of each subplot indicates the LLM name. The matrix color intensities indicate the frequency of predictions, with darker colors representing higher counts.

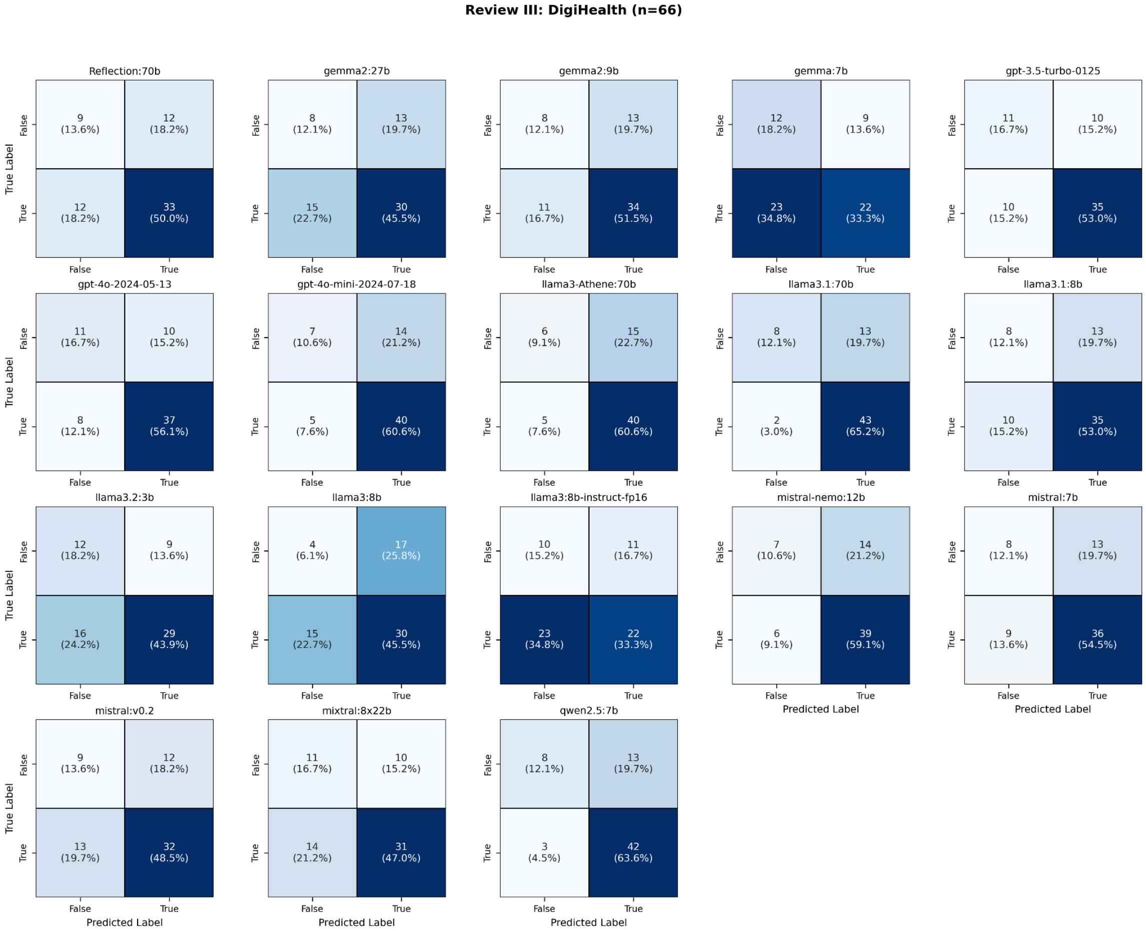

Fig. S6: Confusion Matrices for Models in Review III (DigiHealth) , when an article is predicted as selected (True) using RF. Each subplot represents the confusion matrix of a different model in a given review. The confusion matrices depict the true negative (TN), false positive (FP), false negative (FN), and true positive (TP) classifications of binary labels. The x-axis represents the predicted labels, while the y-axis shows the actual labels. The annotations provide the count of predictions for each quadrant, with the total number of records for each model mentioned in the x-axis label. The title of each subplot indicates the LLM name. The matrix color intensities indicate the frequency of predictions, with darker colors representing higher counts.

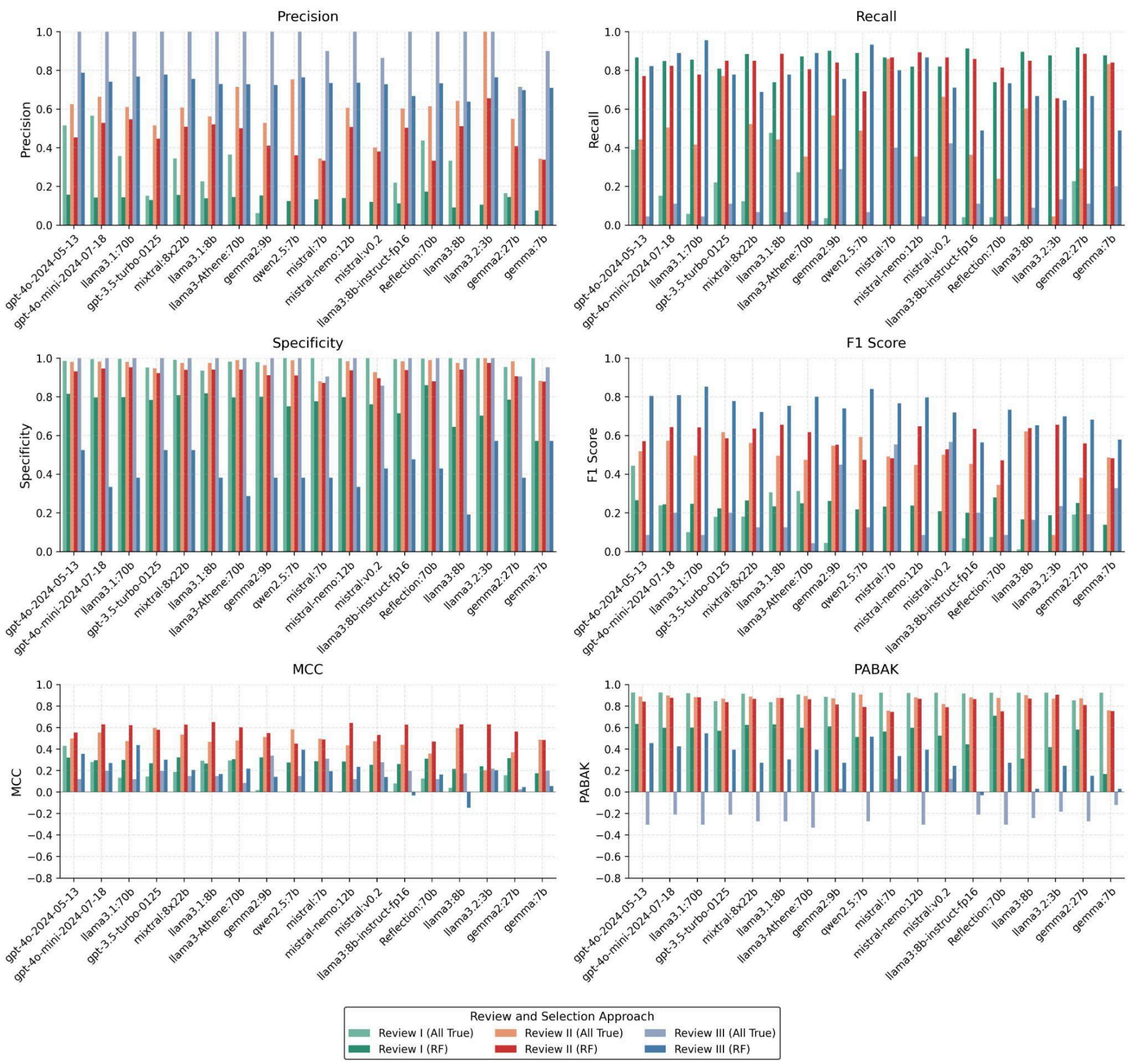

Fig. S7: Comparative Performance Metrics of selecting all criteria as true (all true) vs. Random Forest (RF) models approach across three Systematic Reviews: Review I (Physio) , Review II (Neuro) , and Review III (DigiHealth). Each subplot represents a different evaluation metric—precision, recall, specificity, F1-score, MCC, and PABAK—with the x-axis showing the different models and the y-axis representing the metric values. The bar plots use a color palette to distinguish between the reviews (different colors) and the criteria selection methods (different color brightness).

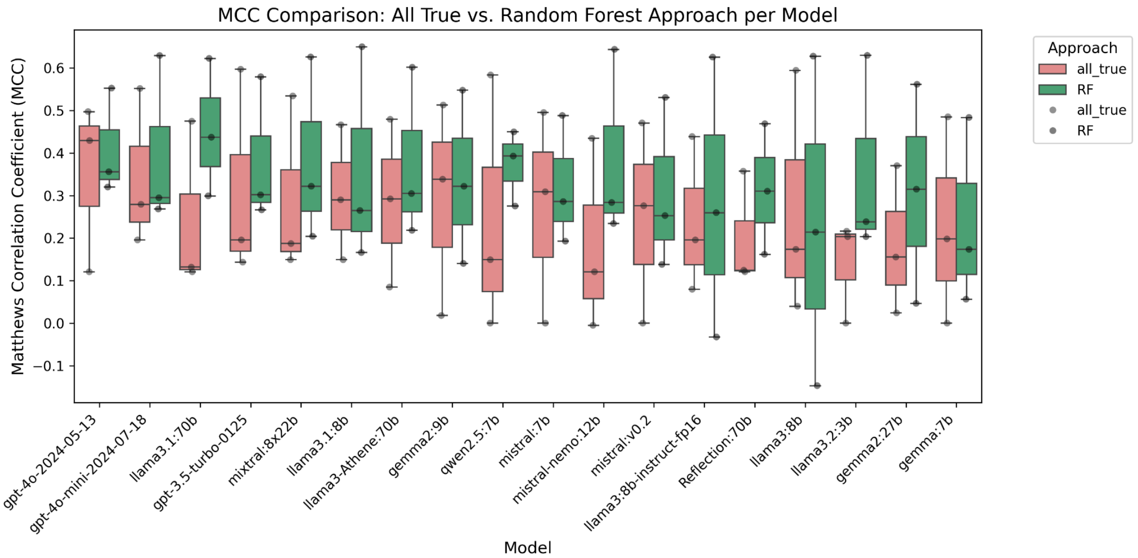

Fig. S8: Boxplot comparing the Matthews Correlation Coefficient (MCC) for the "all true" and Random Forest (RF) approaches across different models. The boxplot displays the distribution of MCC values, with individual data points overlaid for added detail. The comparison highlights how RF performs relative to the "all true" approach for each model, revealing variability in the benefit of using the RF method.
